# High-Intensity Interval Training Combined with High-Load Strength Training Improves Aerobic Fitness, Match Goals and Match Result during the In-Season Period in Under-19 Soccer Players

**DOI:** 10.3390/sports12010002

**Published:** 2023-12-20

**Authors:** Pierros Thomakos, Konstantinos Spyrou, Athanasios Tsoukos, Christos Katsikas, Gregory C. Bogdanis

**Affiliations:** 1 School of Physical Education and Sports Science, National and Kapodistrian University of Athens, 17237 Dafne, Greece; atsoukos@phed.uoa.gr (A.T.); ckatsikas@phed.uoa.gr (C.K.); gbogdanis@phed.uoa.gr (G.C.B.); 2 UCAM Research Center for High Performance Sport, Catholic University of Murcia (UCAM), 30005 Murcia, Spain; kspyrou@ucam.edu; 3 Facultad de Deporte, Catholic University of Murcia (UCAM), 30005 Murcia, Spain

**Keywords:** aerobic fitness training, resistance training, goals scored, match results

## Abstract

This study compared the effects of adding a weekly session of either strength training followed by high-intensity interval training (HIIT) or high load plyometric training followed by small-sided games (SSG) on aerobic fitness, goals scored and conceded, and match results. Twenty-nine players from two youth elite teams competing in an under 19 (U19) first division league took part in the study. Of those, 16 players from one team followed the HIIT program (age: 17.8 ± 0.6 years) and 13 players from the other team trained once a week, following the SSG program (age: 18.0 ± 0.6 years). The training intervention lasted for 11 weeks and was applied once per week. For the HIIT team it included high-load strength training (80–95% of 1 RM) followed by high-intensity intermittent running, whereas for the SSG team (*n* = 13) it included plyometric exercises followed by SSG. Aerobic fitness was assessed before and after the intervention, while goals scored and conceded, as well as match results during the intervention period, were compared with the 11-week period preceding it. Only the HIIT team improved Yo-Yo IR2 performance (running distance: from 902.5 ± 147.5 to 1092.5 ± 155.8 m, *p* < 0.001) while there was no change for the SSG group. Moreover, during the 11-week intervention, the HIIT team improved the number of goals scored in the last 30 min of the game compared with the pre-intervention period (0.36 ± 0.50 to 1.27 ± 0.90 per match, *p* < 0.05) and the scored vs. conceded goals were higher in the second half (1.36 ± 1.03 vs. 0.27 ± 0.47, *p* < 0.05). No significant differences were found for these parameters in the SSG team. Regarding match result, the HIIT team increased the proportion of wins from 5 wins in 11 matches (or 45%) in the pre-intervention period to 9 wins in 11 matches (or 82%) during the intervention period, while this profile remained unchanged for the SSG team. In conclusion, the present study demonstrated that the HIIT program performed once per week was superior to SSG as it improves aerobic fitness, scored and conceded goals, and match result during the in-season period.

## 1. Introduction

The delay of fatigue onset during match play is the main goal of fitness training programs in soccer, as the physical and technical-tactical demands are interrelated [1,2]. When comparing running performance between the three 15-min segments of each half or between the first and second half, there is a significant decline in high-speed running and sprinting [3,4,5,6] and players take longer recovery periods between these actions [7].

Aerobic fitness plays a crucial role in reducing fatigue during match play in soccer. Players with a better aerobic capacity can repeat more intense efforts with shorter recovery intervals and with greater intensity and duration, which also may increase the number of involvements with the ball during match play [8,9]. Notably, top-ranking league teams are characterized by higher aerobic fitness when compared to bottom-ranking league teams [10]. Additionally, Dupont et al. [11] found that high aerobic fitness during the in-season was significantly associated with match performance, i.e., distance covered, level of work intensity, number of sprints, and winning [11]. However, to the authors’ knowledge, no previous study has examined the effects of using two different types of physical training on aspects of match performance such as the number of goals scored and conceded and the match result.

During the in-season period, soccer training aims mainly to maintain physical conditioning while improving technical and mainly tactical aspects [12]. Meanwhile, the frequency and volume of physical fitness and strength training are commonly reduced, as priority is given to drills that enhance tactical-technical aspects to optimize match performance [13,14]. To maintain strength, trainers use plyometric exercises and moderate volume and intensity resistance training programs [15]. However, during the in-season period some trainers employ heavier resistance training programs characterized by low volume and very low frequency, i.e., once per week [15,16,17,18]. Both plyometrics and heavy strength training could improve repeated sprint and endurance performance, muscle power and acceleration, or even running economy [18,19,20,21,22]. High-load resistance training has been shown to be very effective in improving important components of soccer performance such as repeated sprinting ability and running economy [19].

Considering the maintenance or improvement of aerobic and anaerobic fitness, different methods with or without the ball, such as high-intensity interval training (HIIT) or sprint interval training (SIT), and small-sided games (SSG) are commonly employed during the in-season period [1,23,24]. Of these training methods, SSG not only improves high-intensity running performance but also develops technical and tactical aspects related to match-play [23,24]. For example, depending on the number of players, the dimension of the pitch, and the rules employed in SSG, training may be targeted to improve changes of direction, repeated sprint ability, and aerobic-anaerobic capacities [25,26]. On the other hand, running-only approaches, such as HIIT, may provide more control over the individual intensity of training (high-speed running and sprinting), as some players may underperform during SSG [27]. However, running-based approaches may indirectly improve technical or tactical aspects of soccer match-play by increasing muscle oxidative capacity, ion transporters, and antioxidative capacity, in a possibly more effective way in HIIT than in SSG training [28].

Thus, the purpose of the present study was to examine the effects of two different training programs (HIIT versus SSG) applied once per week during the competitive season on (a) aerobic fitness (i.e., total distance, VO2max, and VO2max by Yo-Yo Intermittent Recovery (IR) Test Level 2), (b) goals scored and conceded, and (c) match result (win, draw, loss) during the in-season period for 22 consecutive official matches (11 before and 11 during the intervention). The HIIT program consisted of a combination of high-load, moderate volume resistance training followed by high-intensity intermittent running, while the SSG program consisted of plyometric exercises followed by SSG. Due to the direct and indirect effects of the HIIT program on all players, as described above, we hypothesized that this would have a superior effect on both aerobic fitness and goal scoring in official matches compared with SSG.

## 2. Materials and Methods

### 2.1. Study Design

Twenty-nine soccer players from two youth elite teams competing in the under 19 (U19) 1st division Greek super league took part in the present study. Players from one team (*n* = 16) added a weekly training session consisting of a combination of heavy, moderate volume strength training followed by two 6 min sets of 15 s running at 110% vVO2max, followed by 15 s of passive recovery (HIIT team). On the other days, they trained with the ball using passing games, small-sided games, and tactical games. Players from the other team (*n* = 13) added a weekly training session consisting of a combination of plyometric power training followed (SSG team). The SSG team followed the same training program as the HIIT team and trained with the ball using passing games, SSG, and tactical games. The daily training duration was the same for the SSG and HIIT teams. (Table 1).

The only difference between the HIIT team and the SSG team was that on the second day of the week, the SSG team performed a session including plyometric exercises followed by SSG. The plyometric training program included three sets of bilateral and two sets of unilateral (right and left leg) repeated vertical jumps over 5 hurdles (height 40 cm). The players also performed two sets of 6 horizontal jumps, landing on a Bosu^®^ (BOSU, Ashland, OH, USA) with one leg and jumping again off the other Bosu^®^ to land on the grass. The SSG part of that day included SSG and tactical games (agility/modality for 10 min, SSG for 8 min, and a tactical game for 8 min) (Table 1). Each training session lasted from 50 to 90 min for both teams, including the warm-up and cool-down (Table 1). These interventions were performed during the in-season period, starting in the middle of the second round of the league, for 12 consecutive weeks (end of January until middle of April). The field-tests were performed one week before the start of the intervention (on Tuesday) and in the week after the intervention period (on Wednesday). At the end of the experimental period, the players rested for two days before the test to avoid the effect of any acute or residual fatigue. Before starting the intervention period, the HIIT team was in 7th position in the league and the SSG team was in 10th position in the league. All participants were familiar with the test used as they had been regularly performing them in previous years.

### 2.2. Subjects

Power analysis indicated that a minimum sample size of 22 participants in total would be needed to detect an effect size (ES) of 0.57. This ES was obtained by Clemente et al. (2022), examining the differences between HIIT and SSG during an intervention period in male youth soccer players [29]. Power analysis was performed by using the following parameters: the type of analysis was set to repeated measures mixed ANOVA (within-between subject’s effects), the required power was set to 0.80, alpha was set to 0.05, and the correlation between repeated measures was set to *r* = 0.5 (G-Power software, v.3.1.9.2, Heinrich-Heine-Universität Düsseldorf, Germany). Twenty-nine players from two youth teams (Superleague U19; 1st Division of the Greek league) participated in the present study. Of those, 16 players from one team followed the HIIT program (age: 17.8 ± 0.6 years; body mass: 70.9 ± 6.6 kg; height: 178 ± 0.1 cm; body mass index: 22.4 ± 1.6; and body fat: 7.0 ± 2.7%), and 13 players from the other team followed the SSG (age: 18.0 ± 0.6 years; body mass: 69.9 ± 6.0 kg; height: 177 ± 0.1 cm; body mass index: 22.3 ± 1.9; and body fat: 6.6 ± 2.5%). Goalkeepers, as well as field soccer players who did not train for more than 20% of the total training days due to injuries, or participated in less than 6 league matches, or played for less than 45 min in the matches, were excluded from the study. None of the players received any medication or nutritional supplements and all players signed a written informed consent before entering the research procedure. All procedures were in accordance with the Declaration of Helsinki and were approved by the local university ethics committee (1494/15-03-2023).

### 2.3. Training

This session was performed on the second day of the weekly micro-cycle. During strength training, repetitions were decreased by two per set (starting from 8 to 6 to 4 to 2 repetitions), while the load was gradually increased by 5% (starting from 80 and reaching 95% of 1 RM). The number of sets remained unchanged (i.e., 4 sets) and the recovery between sets was 3 min.

### 2.4. Measurements

Aerobic fitness: Aerobic fitness was evaluated by the Yo-Yo Intermittent Recovery Test Level 2. The test consisted of repeated 2 × 20 m runs back and forth with a progressive increase of running speed (Bangsbosport.com). Between each running bout there was a 10 s active rest period, consisting of 2 × 5 m of jogging. When the participants failed twice to reach the finishing lines, the distance covered was recorded and kept as the test result. The VO2max was estimated for Yo-Yo IR2 test from the following equation [30]: VO2max (mL/min/kg) = IR2 distance (m) × 0.0136 + 45.3.

Scored and conceded goals: The scored and conceded goals in the matches were registered by the technical teams. For the current study, researchers performed a double check of the goals on official website of the Greek Superleague (https://www.slgr.gr/el/ accessed on 29 May 2023) on the youth league U19 championship page. The researchers registered all the scored and conceded goals for 22 official matches (11 matches before and 11 matches during the intervention program performed from October until middle of April). The goals scored during the first and second half, as well as goals scored during the last 30 min of each game (61 until 90+ min), were also recorded and analyzed. Also, the number of wins, draws, and defeats were recorded during the same time periods.

### 2.5. Statistical Analysis

Data are presented as mean ± standard deviations. Statistical analysis was performed using the SPSS (Version 26, Chicago, IL, USA). A mixed-model two-way analysis of variance with repeated measures on one factor (pre and post training) and two teams (HIIT and SSG) was used to examine differences in Yo-Yo IR2 parameters (i.e., total distance, VO2max, vVO2max). The number of scored and conceded goals was also analyzed using mixed-model two-way analysis of variance (ANOVA) with repeated measures on one factor (11 weeks before and 11 matches during the intervention) and a Tukey post-hoc was performed to explore mean differences when a main effect or an interaction was found. Separate mixed model two-way ANOVAs were conducted to compare the scored and conceded goals before and during the intervention in the first and second halves. Cohen’s effect sizes (ESs) with 95% confidence intervals (95% CIs) were computed to determine the magnitude of every paired comparison and classified as follows: trivial (<0.2), small (>0.2–0.6), moderate (>0.6–1.2), large (>1.2–2.0), and very large (>2.0–4.0). The level of significance was set at *p* < 0.05.

## 3. Results

**Figure 1:**
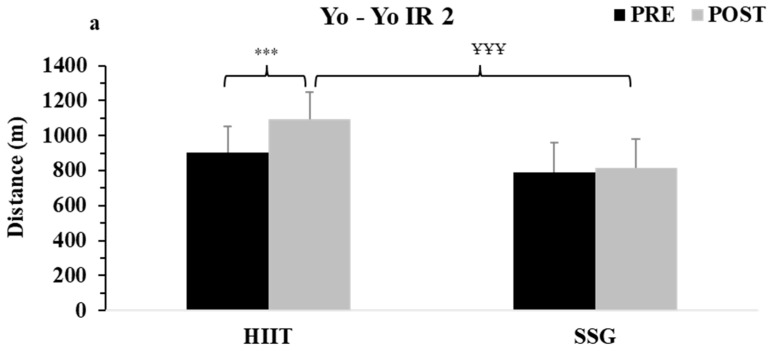

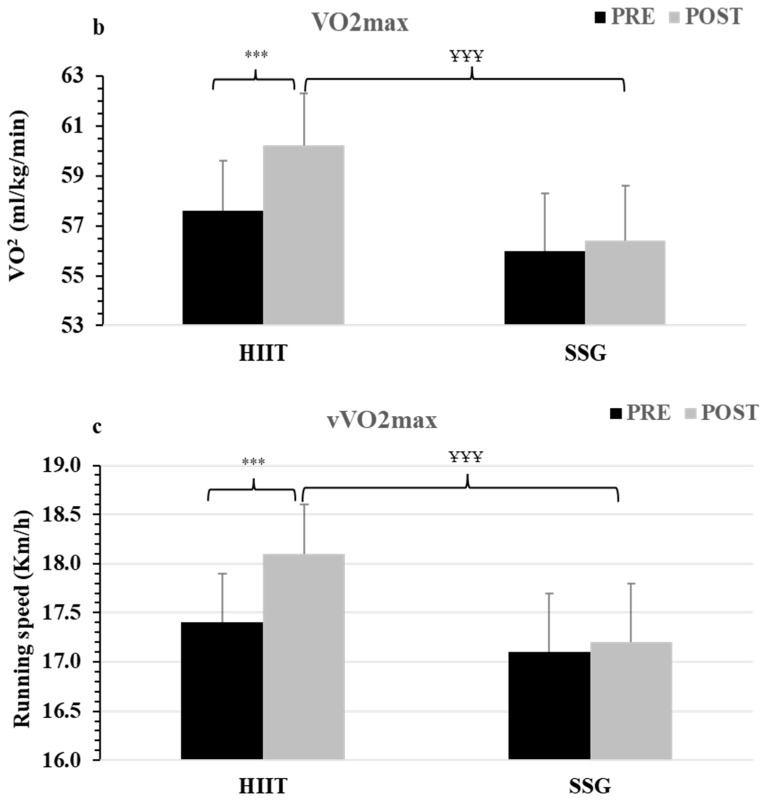
(**a**) Distance covered in Yo-Yo IR2 expressed in meters (m); (**b**) Calculated maximum oxygen uptake (VO2max) expressed in mL/kg/min; (**c**) Running speed corresponding to VO2max (vVO2max) expressed in km/h; HIIT: high intensity interval training team; SSG: small-sided games team; PRE with black column and POST with gray column; *** *p* < 0.001 between pre and post at HIIT team; ^ҰҰҰ^ *p* < 0.001 at post between HIIT and SSG teams.

No significant differences were found in aerobic parameters before the intervention period between the two teams (HIIT and SSG) (distance covered: 902.5 ± 147.5 vs. 787.7 ± 172.3 m; VO2max: 57.6 ± 2.0 vs. 56.0 ± 2.3 mL/kg/min; vVO2: 17.4 ± 0.5 vs. 17.1 ± 0.6 km/h). Aerobic fitness assessed by distance run, VO2max, and running speed at VO2max improved after the intervention only in the HIIT group (running distance: from 902.5 ± 147.5 to 1092.5 ± 155.8 m, *d* = 1.25 large; VO2max: 57.6 ± 2.0 vs. 60.2 ± 2.1 mL/kg/min, *d* = 1.27 large, running speed: 17.4 ± 0.5 vs. 18.1 ± 0.5 km/h/*d*: 1.4 large, *p* = 0.0002). As shown in Figure 1a–c, aerobic fitness parameters remained unchanged in the SSG team. Also, the post-intervention values were higher in the HIIT team compared to the SSG team (*p* < 0.001, *d* = 1.63 to 1.77, large).

Table 2 and Table 3 present the scored and conceded goals by the two teams before and during the intervention period. There were no significant differences in scored and conceded goals between teams in the 11 matches before the intervention. The HIIT team demonstrated an increase in the number of scored goals in the last 30 min of the game during the intervention period compared with the pre-intervention period (0.36 ± 0.50 to 1.27 ± 0.90 goals per match, *p* = 0.040; *d* = 1.25, large). Also, the scored versus conceded goals were higher in the second half (1.36 ± 1.03 vs. 0.27 ± 0.47 respectively, *p* = 0.005; *d* = 1.36, large) and during the last 30 min in HIIT (1.27 ± 0.90 vs. 0.18 ± 0.40 respectively, *p* = 0.005; *d* = 1.57, large) (Table 2). No significant differences were found for the SSG scored versus conceded goals both before and during the intervention period (Table 3).

The number and percentage of wins, draws, and defeats were similar before the intervention period in both the HIIT and the SSG teams (5 wins—45%, 1 draw—9%, 5 defeats—45% vs. 4 wins—36%, 3 draws—27%, 4 defeats—36%, respectively). However, during the intervention the match results changed for the HIIT team in favor of wins (9 wins—82%, 1 draw—9%, 1 defeat—9%), while it remained unchanged for the SSG team (3 wins—27%, 3 draws—27%, 5 defeats—45%). Lastly, the HIIT team finished in 3rd position while the SSG team finished in 9th position, while before the study the HIIT team was 7th and the SSG team was 10th.

## 4. Discussion

The aim of this study was to examine and compare the effects of HIIT and SSG on aerobic fitness, match goals, and match result during the in-season period in U19 youth soccer players. Two different teams performed two training programs, one based on resistance training combined with HIIT once per week (HIIT team), while the other team trained only with the ball and used plyometric exercises in combination with tactical and SSG on the same day of weekly plan (SSG team). The main finding was that Yo-Yo IR2 performance was improved for the HIIT team, while it remained unchanged for the SSG team. Moreover, this increase in intermittent aerobic performance was coupled with an increase in the number of wins, goals scored, and a reduction in the number of goals conceded in the second part of the game, particularly during the last 30 min, only for the HIIT team. Notably, there were no changes in aerobic fitness or goals scored or conceded for the SSG team during the 11 weeks of intervention.

Aerobic fitness showed a significant improvement for the HIIT team, while it remained unchanged for the SSG team. This finding is in line with previous studies comparing HIIT with SSG during the in-season period [23,24,31] and may be explained by the superior metabolic adaptations (i.e., increase in oxidative capacity and ion transporters) in all players following similar high-intensity running approaches compared with SSG [28]. During the in-season period, most coaches use SSG because they contain technical and tactical elements that may be directly applied in the game [11,23]. However, some researchers implemented HIIT or SIT to achieve further adaptations that are important for match performance [1,28,32]. For example, Dupont et al. [11] compared a control period consisting of technical and tactical skills, games, and matches with an experimental period which included very high intensity 15 s running and 15 s passive rest at 120% of VO2max during the in-season. The main finding was that the team won 33.3% of its games during the control period and 77.8% of its games during the HIIT period, which is similar with the findings of the present study (45% vs. 82% wins, respectively). In the present study, the intervention program containing resistance training using heavy loads was followed by 2 × 6 min of 15 s–15 s intermittent running at 110% of vVO2max, while the next day included a technical-tactical program with small and medium sized games, which aimed to control the workload and reduce the likelihood of fatigue on match-day [26,33]. The team following the 11-week heavy resistance and high-intensity running intervention had a large improvement in match outcome (i.e., more wins) and demonstrated an increase in the number of goals scored in the second half and especially in the last 30 min of the match, while the number of conceded goals was reduced compared to the pre-intervention period. In contrast, during the same period, the SSG team showed no changes in the match outcome or the scored and conceded goals. Thus, the combination of HIIT and heavy, moderate volume resistance training may be superior to the plyometrics and SSG approach when the training frequency is once per week.

During the in-season period, the main aim is to improve technical-tactical performance and consequently match play, using mainly games and related soccer drills. Training containing SSG has been shown to be effective in improving aerobic fitness in players with a low initial level [34], as well as in elite youth soccer players [24]. In the present study, the difference between the two teams was on the day in which the HIIT team performed strength training and high-intensity aerobic running, while the SSG team performed plyometric training followed by small sided and tactical games. One clear advantage for the HIIT team was the large improvement in intermittent running aerobic performance, assessed by the Yo-Yo IR2 test. A previous study by Mohr & Krustrup (2016) [31]. compared intervention programs containing speed endurance production training (30 s work with 2–3 min rest between sets) and speed endurance maintenance training involving SSG (2 vs. 2–4 vs. 4) with 30–60 s rest. The results showed a higher improvement in endurance after the speed endurance production training [31] which is in agreement with the findings of the present study. One additional benefit of combining heavy load, moderate volume resistance training before high-intensity training is the possible maintenance or improvement of strength [21]. However, due to practical issues (i.e., refusal of the coaches to perform maximal strength tests during the in-season), we could not measure any changes in strength. Although this is a limitation of the present study, it may be argued that in-season resistance strength training with high intensity, moderate volume training performed once per week maintained the level of strength and power achieved during the pre-season [14,33,35] which would otherwise decline [36]. This approach of strength and power maintenance may be superior to plyometric training during the in-season period [15]. Furthermore, the combination of strength and endurance training has been shown to improve endurance performance to a greater degree than endurance training alone [37]. For example, Balabinis et al. [38] showed that the combination of strength and endurance training improved VO2max by 12.9%, whereas the improvement was lower (6.8%) for the endurance only training group. This difference in VO2max improvement may be related to the improvement in running economy.

The goals scored compared to the goals conceded in the second half and during the last 30 min of the game were higher in the HIIT team (*p* = 0.018). Match analyses have shown that in the second half and mainly in the last 15 min, the performance of the players decreases, and this is demonstrated by the lower total distance covered by high-intensity running, the lower number of sprints, and the increased recovery time required between high-intensity actions [1,5,6,7,39]. Thus, the present study confirms that improved aerobic fitness as assessed by YoY o IR2 performance is associated with improved match performance [11,40] as shown by the almost double number of wins compared with the pre-intervention period (82% vs. 45%) and the large increase in the goals scored, especially towards the end of the game. Moreover, the HIIT team finished in 3rd position while the SSG team finished in 9th position while before the study the position of the HIIT team was 7th and the SSG team was 10th. Helgerud et al. [8] showed that by increasing VO2max, running economy, and lactate threshold, the distance covered during the game increased by 20%, the number of sprints increased by 100%, and the number of involvements with the ball increased by 24%. In the present study, the Yo-Yo IR2 improved significantly in the HIIT team while it remained unchanged in the SSG team. Interestingly, Mohr & Krustrup [10] found that in the Danish semi-professional soccer league, the top-teams had 18% higher Yo-Yo IR2 performance in comparison to bottom-teams at pre-season and this remained higher at all testing points during the in-season.

The present study has some limitations. Due to technical and logistic issues, external load data (e.g., GPS data) were not available. This could have provided further details regarding the load of the training and match analysis, and the increase in running performance as a result of an increased Yo-Yo IR2 performance. In addition, strength-power tests were not obtained because coaches did not want the players to be tested during the in-season. Furthermore, part of the improvement in total distance in the Yo-Yo IR2 test might be due to an increase in running economy. Also, we have no data regarding the effects of other factors such as the technical and tactical abilities of the players on goal scoring and on team performance [37,41,42]. Of course, we could not overlook the complex nature of soccer performance and goal scoring which may be influenced by many other factors such as technical, tactical, and mental aspects, the opponent teams, and the randomness in scoring goals. However, the lack of improvement in goal scoring, soccer results, and position of the SSG team in the league were not affected by training, lending support to the effectiveness of the training followed by the HIIT team. Nevertheless, this approach should be further tested in other teams and age groups in professional soccer.

In conclusion, the current study showed that in-season resistance and HIIT training once per week improved intermittent endurance performance and this influenced goal scoring and match outcome. Importantly, the increase in the goals scored during the second half and especially during the last 30 min of the match may indicate reduced fatigue and improved running performance as a result of the HIIT training compared to the SSG training intervention.

## Figures and Tables

**Table 1:** Description of the two training programs during the week.

HIIT Team								
Day of Week	1	2		3	4	5	6	7
Type	Ball Training	Resistance	Interval	Ball Training	Ball Training	Ball Training	Match day	Day off
Exercises	(i) Passing games	(i) Bench press	15 s/15 s	(i) Agility/mobility	(i) Agility/mobility	(i) Agility/mobility		
	(ii) Small-sided games	(ii) Half-squat	15 s Running	(ii) Passing games	(ii) Small-sided games	(ii) Passing games		
	(iii) Tactical games	(iii) Hip-trust	15 s Passive	(iii) Small-sided games	(iii) Tactical games	(iii) Tactical games		
		(iv) Leg curl	Recovery	(iv) Tactical games				
		(v) Leg extension						
Sets/Reps	(i) 2	1/8	2	(i) 1	(i) 1	(i) 1		
	(ii) 4	1/6		(ii) 2	(ii) 2	(ii) 2		
	(iii) 2	1/4		(iii) 2	(iii) 3	(iii) 2		
		1/2		(iv) 3				
Duration/Intensity	(i) 8 min	80% 1 RM	6 min	(i) 10 min	(i) 10 min	(i) 8 min		
	(ii) 4 min	85% 1 RM	110% vVO2max	(ii) 6 min	(ii) 6 min	(ii) 4 min		
	(iii) 8 min	90% 1 RM		(iii) 6 min	(iii) 8 min	(iii) 5 min		
		95% 1 RM		(iv) 8 min				
Rest	3 min RBS	3 min RBS	3 min RBS	2 min RBS	3 min RBS	3 min RBS		
Total Duration	90 min	90 min		90 min	75 min	50 min		
**SSG Team**								
**Day of week**	**1**	**2**		**3**	**4**	**5**	**6**	**7**
Type	Ball Training	Ball Training		Ball Training	Ball Training	Ball Training	Match day	Day off
Exercises	(i) Passing games	(i) Agility/mobility		(i) Agility/mobility	(i) Agility/mobility	(i) Agility/mobility		
	(ii) Small-sided games	(ii) Plyometric exercises		(ii) Passing games	(ii) Small-sided games	(ii) Passing games		
	(iii) Tactical games	(iii) Small-sided games		(iii) Small-sided games	(iii) Tactical games	(iii) Tactical games		
		(iv) Tactical games		(iv) Tactical games				
Sets/Reps	(i) 2	(i) 1		(i) 1	(i) 1	(i) 1		
	(ii) 4	(ii) 1		(ii) 2	(ii) 2	(ii) 2		
	(iii) 2	(iii) 2		(iii) 2	(iii) 3	(iii) 2		
		(iv) 3		(iv) 3				
Duration/Intensity	(i) 8 min	(i)10 min		(i) 10 min	(i) 10 min	(i) 8 min		
	(ii) 4 min	(ii) 15 min		(ii) 6 min	(ii) 6 min	(ii) 4 min		
	(iii) 8 min	(iii) 8 min		(iii) 6 min	(iii) 8 min	(iii) 5 min		
		(iv) 8 min		(iv) 8 min				
Rest	3 min RBS	3 min RBS		2 min RBS	3 min RBS	3 min RBS		
Total Duration	90 min	90 min		90 min	75 min	50 min		

Small-sided games included 4 vs. 4 up to 6 vs. 6 players. Tactical games included 7 vs. 7 up to 10 vs. 10 players. RBS: Recovery between sets; HIIT: High Intensity Interval Training; SSG: small-side games.

**Table 2:** Description of the scored and conceded goals in the HIIT team.

HIIT Team					Goals Scored						Goals Conceded					
Period	Match Time (min)		Score	Result	1–15	16–30	31–45	46–60	61–75	76–90+	1–15	16–30	31–45	46–60	61–75	76–90+
**PRE**	Games	1 (H)	1-2	L			1				1			1		
		2 (A)	1-0	L												1
		3 (H)	2-1	W		1	1									1
		4 (A)	3-0	L									1		1	1
		5 (H)	4-3	W	1	1		1		1				1		2
		6 (A)	1-0	L										1		
		7 (H)	1-3	L		1					1					2
		8 (A)	0-2	W			1		1							
		9 (A)	2-3	W		1	1	1				1	1			
		10 (A)	1-2	W				1	1					1		
		11 (H)	1-1	D						1	1					
	Goals				**Total goals 1st Half**			**Total goals 2nd Half**			**Total goals 1st Half**			**Total goals 2nd Half**		
					9			7			6			12		
		**Total goals 1st + 2nd**			16						18					
		**Mean ± SD**			0.82 ± 0.87			0.64 ± 0.81			0.55 ± 0.69			1.09 ± 1.04		
		**Mean ± SD 61–90+:**			0.36 ± 0.50 */*d*: 1.25 (Large)						0.73 ± 0.90					
**POST**	Games	1 (H)	5-2	W	1	2	1			1		1		1		
		2 (H)	1-0	W					1							
		3 (A)	1-2	W	1					1						1
		4 (H)	1-0	W			1									
		5 (A)	2-1	L						1		1			1	
		6 (H)	3-0	W					1	2						
		7 (A)	0-1	W						1						
		8 (A)	0-0	D												
		9 (H)	4-0	W			1	1	1	1						
		10 (A)	2-4	W		1	1			2		2				
		11 (H)	2-1	W						2		1				
	Goals				**Total goals 1st Half**			**Total goals 2nd Half**			**Total goals 1st Half**			**Total goals 2nd Half**		
					9			15			5			3		
		**Total goals 1st + 2nd**			24						8					
		**Mean ± SD**			0.82 ± 1.25			1.36 ± 1.03 ^ҰҰ^ *d*:1.36 (Large)			0.45 ± 0.69			0.27 ± 0.47		
		**Mean ± SD 61–90+:**			1.27 ± 0.90 ^ҰҰ^/*d*:1.57 (Large)						0.18 ± 0.40					

A: Away; D: draw; H: Home; HIIT: High Intensity Interval Training; L: loss; SD: Standard Deviation; W: win; * *p* < 0.05 statistically significant difference to scored goals between pre and post from 61 min onwards until the end of match; ^ҰҰ^ *p* < 0.01 statistically significant difference between scored and conceded goals in second half, also from 61 min onwards until the end of match, at post.

**Table 3:** Description of the scored and conceded goals from the SSG team.

SSG Group					Goals Scored						Goals Conceded					
Period	Match Time (Min)		Score	Result	1–15	16–30	31–45	46–60	61–75	76–90+	1–15	16–30	31–45	46–60	61–75	76–90+
**PRE**	Games	1 (A)	2-0	L									1	1		
		2 (H)	5-0	W		1	1	1	1	1						
		3 (A)	4-1	L				1			2		1		1	
		4 (A)	1-1	D			1							1		
		5 (H)	1-1	D						1			1			
		6 (A)	1-2	W					1	1						1
		7 (H)	0-2	L									1		1	
		8 (A)	1-3	W	1			1	1			1				
		9 (H)	1-1	D						1	1					
		10 (A)	1-2	W	1				1							1
		11 (A)	1-0	L							1					
	Goals				**Total goals 1st Half**			**Total goals 2nd Half**			**Total goals 1st Half**			**Total goals 2nd Half**		
					5			11			9			6		
		**Total goals 1st + 2nd**			16						15					
		**Mean ± SD**			0.45 ± 0.69			1.00 ± 1.00			0.82 ± 0.87			0.55 ± 0.52		
		**Mean ± SD 61–90+:**			0.73 ± 0.79						0.36 ± 0.50					
**POST**	Games	1 (A)	2-1	L	1								1			1
		2 (H)	1-1	D				1								1
		3 (A)	1-3	W	1	1			1						1	
		4 (H)	1-2	L						1		1				1
		5 (H)	2-0	W			1			1						
		6 (A)	1-1	D						1		1				
		7 (H)	1-2	L	1								1			1
		8 (A)	2-0	L								1		1		
		9 (H)	1-1	D						1			1			
		10(A)	2-1	L	1						1					1
		11(H)	2-0	W		1		1								
	Goals				**Total goals 1st Half**			**Total goals 2nd Half**			**Total goals 1st Half**			**Total goals 2nd Half**		
					7			7			7			7		
		**Total goals 1st + 2nd**			14						14					
		**Mean ± SD**			0.64 ± 0.67			0.64 ± 0.50			0.64 ± 0.50			0.64 ± 0.50		
		**Mean ± SD 61–90+:**			0.45 ± 0.52						0.55 ± 0.52					

A: Away; D: draw; H: Home; L: loss; SD: Standard Deviation; SSG: Small-Sided Games team; W: win.

## Data Availability

The data are available upon request to the corresponding author due to the football teams’ data privacy policy.
